# Ramularia leaf spot: PCR-based methods reveal widespread distribution of *Ramulariopsis pseudoglycines* and limited presence of *R. gossypii* in Brazil

**DOI:** 10.1038/s41598-023-33530-3

**Published:** 2023-06-17

**Authors:** Aline Suelen da Silva, Marcelo Henrique Lisboa Rennó, Ana Clara Ribeiro Quitania, Adalberto Corrêa Café-Filho, Robert Neil Gerard Miller, Alderi Emidio de Araújo, Danilo Batista Pinho

**Affiliations:** 1 Universidade de Brasília, Brasília, DF 70910-000 Brazil; 2 Embrapa Algodão, Campina Grande, PB 58428-095 Brazil

**Keywords:** Infectious-disease diagnostics, Pathogens

## Abstract

Whilst Brazil is the fourth largest cotton producer globally, incidence of ramularia leaf spot (RLS) has decreased yield. In 2017–18 and 2018–19, ca. 300 fungal samples were collected throughout Brazil. Hyphal tip cultures were obtained for amplification of the RNA polymerase II (*RPB2)*, 28S rRNA, the ribosomal DNA internal transcribed spacers (*ITS*), actin (*ACT)*, elongation factor (*EF1-α*) and histone H3 (*HIS3)* genomic regions. Additionally, sequences of the glyceraldehyde-3-phosphate dehydrogenase (GAPDH) were obtained by nanopore sequencing and the *EF1-α* region was selected as a marker for rapid recognition of *Ramulariopsis* species. Clade assignments based on the concatenated-sequence tree were identical to those in tree generated by *RPB2*-sequences, as well as in an *RPB2* haplotype network and an ISSR (TGTC)^4^ dendrogram, in identification with species-specific primers and based on morphological comparisons. Out of 267 examined isolates, 252 were identified as *Ramulariopsis pseudoglycines*, indicating this species as the most widespread causal agent of cotton RLS in the Brazilian growing regions. Species-specific primers developed in the study that target the *EF1-α* gene provide an opportunity for extensive RLS sampling worldwide to study the distribution of *Ramulariopsis* species. Such data will aid breeders and plant pathologists in cotton disease resistance development and fungicide resistance avoidance.

## Introduction

Cotton (*Gossypium* spp.) is the world´s most cultivated fibre crop, mostly for the supply of raw materials for the textile industry, as well as for oil and protein extraction^1^. Since the 1990s, Brazil has ranked globally in fourth place in terms of cotton production, following strong investments in the production technology and expansion of cultivated areas, mainly in the Brazilian Cerrado biome^2–5^ (a savannah-like region in the Brazilian Midwest).

Favorable environmental conditions during the growing season, combined with the cultivation of a limited number of cotton genotypes over very large areas, favour epidemics of ramularia leaf spot^4,6^. This disease was first reported in Paraguari, Paraguay in 1883^7^, and soon after was followed by a report from Alabama, USA in 1890^8^. Since then, the disease has been reported in more than 40 cotton-producing countries^9,10^. Typically, ramularia leaf spot (RLS) is observed at the end of the cotton cycle and has therefore generally been considered a disease of secondary importance.

The disease was first reported in Brazil by the Agricultural Inspection and Defense Service of São Paulo in 1919 and was considered of secondary importance until the 1990s^9–11^. Currently, RLS is the major cotton disease in Brazil, with up to eight fungicide spray applications required to reduce the negative effects on yield and cotton fibre quality^4,5,12^.

Historically, *Ramularia areola* was the specific epithet used to designate the causal agent of RLS^13^. In 1961, this species was recombined to *Ramularia gossypii* (Speg.) Cif. and, due to the morphological similarities with the related genus *Ramulariopsis* (conidiophores severely branched at the base with terminal and lateral conidiogenic cells), the taxon was recombined again in 1993 to *Ramulariopsis gossypii* (Speg.) Braun^14^. Later, a multigenic study including a representative number of isolates of *Ramularia* and allied genera revealed a new species, *Ramulariopsis pseudoglycines*, associated with RLS^15^.

Up to now, the etiology of the disease in Brazil has remained inaccurate, given that most identifications are based on only few isolates or limited to the examination of morphological data^15–17^. Furthermore, it has been shown that a molecular perspective, associated with morphological data, is required to resolve plant pathogen species complexes, with this combined approach effective in revealing previously uncharacterized species affecting different crops^15,18,19^. For example, molecular characterization of isolates previously identified as *Ramularia eucalypti* based exclusively on morphological comparisons revealed a total of seven species associated with the eucalyptus leaf spot^20^.

Although the sequencing of target regions is efficient for the accurate identification of *Ramulariopsis* species, recommended GAPDH sequences as secondary barcodes for identification of *Ramularia* and allied genera are currently absent for *R. pseudoglycines* in public databases^15,20^. Furthermore, sequencing-based approaches for molecular identification are time-consuming, costly and dependent upon trained personnel with expertise in phylogenetic analysis compared to simple visualization of amplicons obtained with species-specific primers^21^. As such, more accessible specific and rapid molecular tools are needed for routine identification of *R. gossypii* and *R. pseudoglycines*.

The diverse reactions of resistant and susceptible genotypes of cotton to different isolates of *Ramulariopsis* have been ascribed to the genetic variability of the pathogen^22^. An analysis of the genetic diversity of 16 isolates of *Ramulariopsis* previously revealed three genetic groups^23^. Such information indicates a greater variability of the causal agent of the RLS than has so far been acknowledged. Nevertheless, there are still relatively few studies addressing the genetic diversity of *Ramulariopsis* associated with cotton in Brazil^4,22–25^.

Accurate identification of the RLS pathogen in Brazil, as well as determination of the relative abundancy of each causal agent, and respective geographic distribution, are particularly important for disease management approaches, and are paramount for cotton breeding programs. The precise identification of the *Ramulariopsis* species in each growing area, combined with appropriate phytosanitary measures, can minimize damage caused by RLS.

Here, *Ramulariopsis* isolates collected in the main cotton-growing regions in Brazil were morphologically and molecularly characterized. In addition, a PCR-based method was developed to distinguish between isolates of *R. gossypii* and *R. pseudoglycines*, which will aid breeders and plant pathologists in cotton disease resistance development and fungicide resistance avoidance.

## Results

### Sampling and isolates

**Figure 1:**
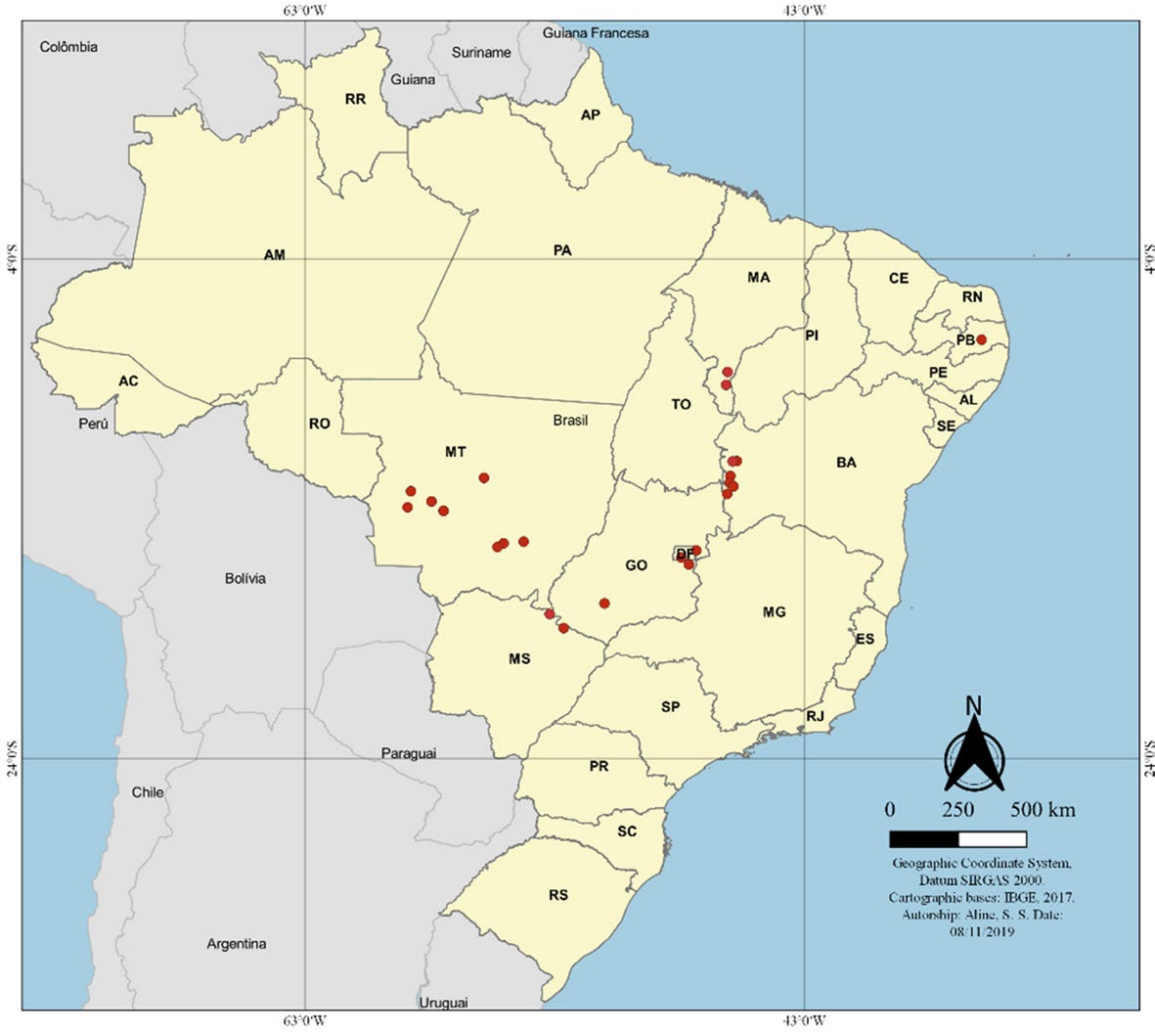
Map of Brazil showing the distribution of states and collection points of *Ramulariopsis* isolates.

**Figure 2:**
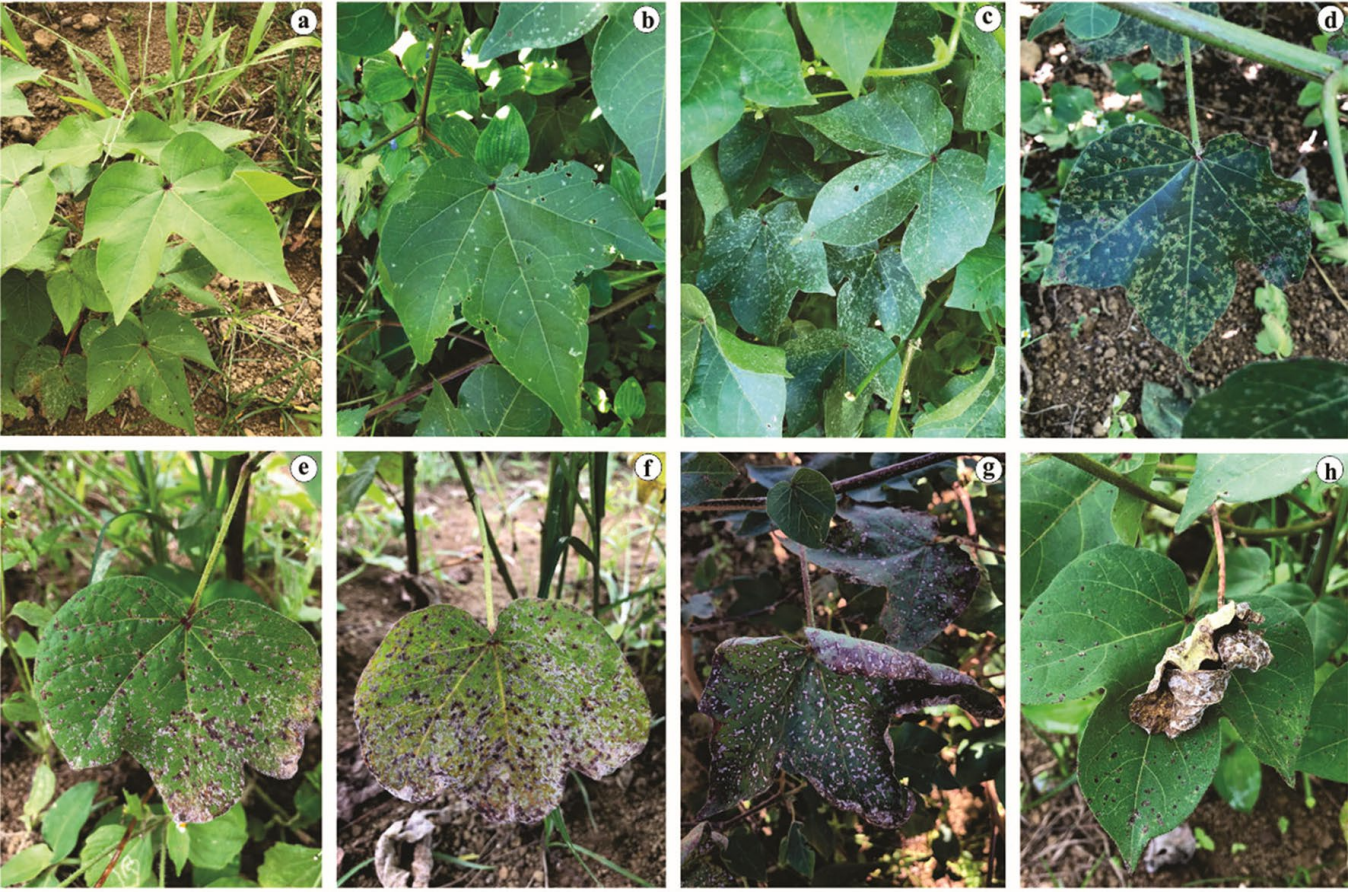
**(a–h)** Ramularia leaf spot symptoms in cotton. (**a**) Healthy leaves observed in the field. (**b, c, d**) Initial symptoms; Early sporulation of *Ramulariopsis* on adaxial sides of cotton leaf; (**e, f**) Late sporulation on necrotic lesions on the upper surface of cotton leaf; (**g**) Necrotic lesions covering the leaf. (**h**) Collapsed leaf with advanced necrotic lesions.

Symptomatic leaf samples were collected from 24 growing fields representing seven Brazilian states (Fig. 1). Naturally occurring symptoms included light green to yellow-green lesions delimited by the veinlets, giving them an angular or irregular shape, with white powdery sporulation on both sides of the leaves. Under favourable disease conditions, lesions coalesced, become chlorotic and then necrotic, often resulting in severe defoliation (Fig. 2). A total of 267 *Ramulariopsis* isolates (Supplementary Table S1) were obtained in the Brazilian states of Bahia (n = 44), Distrito Federal (n = 32), Goiás (n = 31), Maranhão (n = 30), Mato Grosso (n = 87), Mato Grosso do Sul (n = 39) and Paraíba (n = 4). The voucher specimens of *R. gossypii* and *R. pseudoglycines* were deposited at the Herbarium of the University of Brasilia (UB).

### Phylogenetic analysis

**Figure 3:**
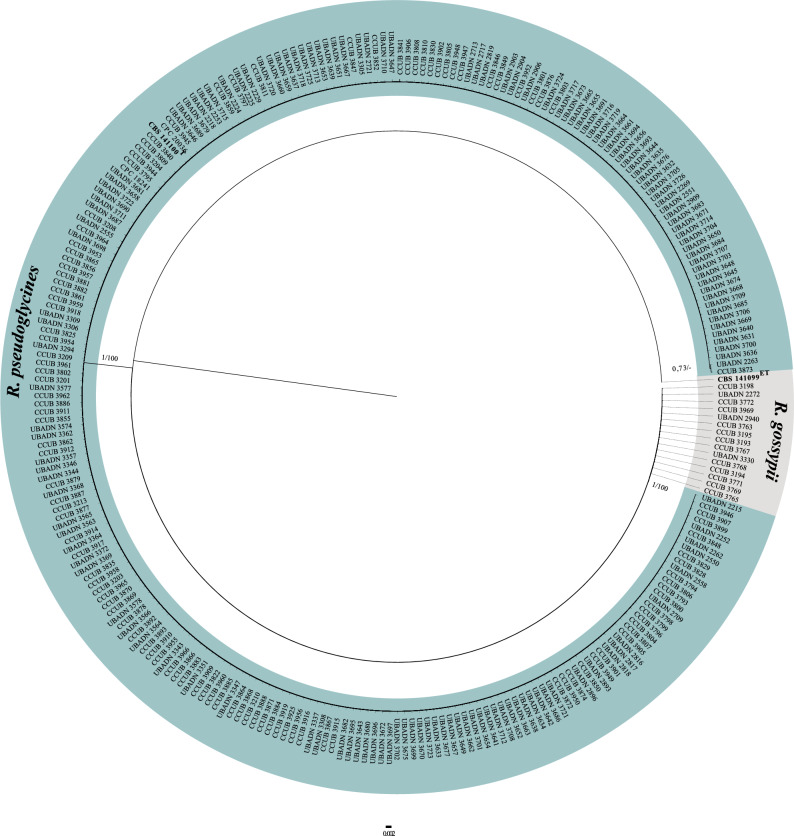
Bayesian phylogenetic tree based on *RPB2* sequences of *Ramulariopsis* species. Bayesian posterior probabilities (BPP) and Maximum Likelihood bootstrap support values (MLBS) are indicated at the nodes (BPP/MLBS), and the scale bar represents the number of expected changes per site. Ex-type isolates are highlighted in bold. The ex-type CBS 141099 of *Ramulariopsis gossypii* was used as outgroup.

*RPB2* amplicons were obtained for all 267 isolates, generating sequences of approximately 930 bp, which were deposited in GenBank under accession nos. MZ039858 to MZ040124. The *RPB2* matrix included 271 taxa (267 isolates from this study and 4 taxa from GenBank), composed of 847 sites (740 conserved) and 91 parsimony-informative characters. The BI tree was reconstructed using the GTR nucleotide substitution model. The *RPB2* tree (Fig. 3) showed that the *Ramulariopsis* isolates were grouped into two distinct clades (the nucleotide matrices and phylogenetic tree are available in TreeBASE; study number S28159). Clade II gathered most (94.4%) of the isolates from the states of Bahia (44), Distrito Federal (21), Goiás (31), Maranhão (30), Mato Grosso (87) and Mato Grosso do Sul (39). The remaining 15 isolates (5.6%) were grouped in Clade I, with 11 isolates from the Distrito Federal and four from the state of Paraíba.

**Figure 4:**
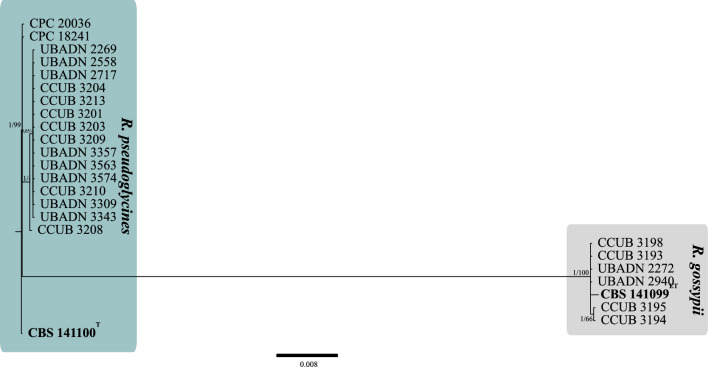
Bayesian phylogenetic tree based on concatenate sequences (*RPB2*, *LSU*, *EF1-α*, *ITS*, *ACT*, and *HIS3*) of *Ramulariopsis* species. Bayesian posterior probabilities (BPP) and Maximum Likelihood bootstrap support values (MLBS) are indicated at the nodes (BPP/MLBS), and the scale bar represents the number of expected changes per site. Ex-type isolates are highlighted in bold. The type CBS 141100 of *Ramulariopsis pseudoglycines* was used as outgroup.

The GAPDH sequences obtained by nanopore sequencing were 571 and 636 bp in length for *R. gossypii* and *R. pseudoglycines*, respectively. The GAPDH sequence of *R. pseudoglycines* showed 135 single nucleotide polymorphisms compared to *R. gossypii* sequences. The GAPDH sequences of *Ramulariopsis gossypii* obtained by nanopore sequencing were identical to the sequences obtained by Sanger sequencing. To correctly delimit the *Ramulariopsis* isolates at the species level, a multilocus approach was adopted using the *RPB2*, *LSU*, *EF1-α*, *ITS*, *ACT*, and *HIS3* sequences. A total of 21 taxa (Supplementary Table S2) were included in the BI and ML phylogenetic analyses. The *RPB2*, *LSU*, *EF1-α*, *ITS*, *ACT*, and *HIS3* individually aligned data sets were 942, 873, 1112, 182, 156, and 346 bp in length, respectively (single gene trees are available in TreeBASE; study number S28159). The concatenate alignment comprised 3611 characters, with 3316 and 281 conserved and variable sites, respectively. Also, 279 sites were determined as phylogenetically informative. The Bayesian phylogenetic tree was reconstructed considering the best nucleotide substitution model for each partition in the concatenate data, GTR (*RPB2*), HKY (*EF1-α, HIS3*, *ITS*, LSU) and K80 (*ACT*). The *Ramulariopsis* isolates reported here were grouped into two distinct phylogenetic clades (Fig. 4), corresponding to *R. gossypii* (clade I) and *R. pseudoglycines* (clade II).

### Primer design and validation

**Figure 5:**
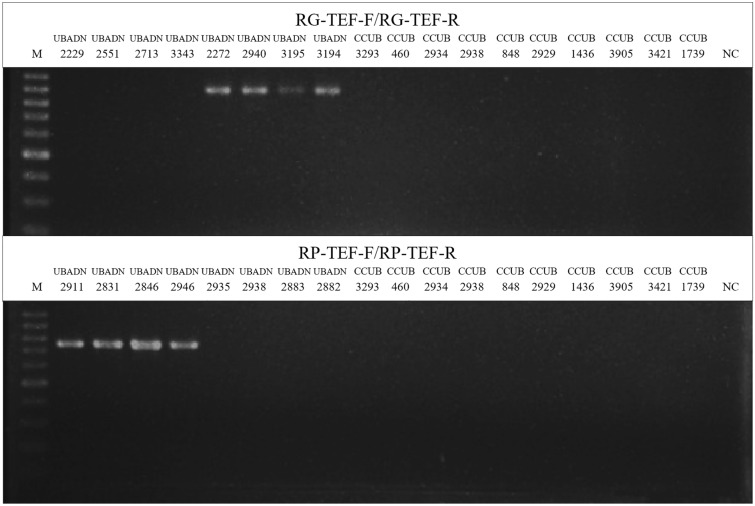
Amplicons obtained using RG-TEF-F/RG-TEF-R and RP-TEF-F/RG-TEF-R primers and visualized on 1.5% agarose gels for isolates of *R. pseudoglycines* (UBADN 2229, UBADN 2551, UBADN 2713, UBADN 3343), *R. gossypii* (UBADN 2272, UBADN 2940, CCUB 3195, CCUB 3194), *Fusarium* sp. (CCUB 3293), *Colletotrichum* sp. (CCUB 460), *Talaromyces* sp. (CCUB 2934) and *Baudoinia* sp. (CCUB 2938), *Cercospora* sp. (CCUB 848), *Aspergillus* sp. (CCUB 2929), *Lasiodiplodia* sp. (CCUB 1436), *Macrophomina* sp. (CCUB 3905), *Trichoderma* sp. (CCUB 3421) and *Phytophthora* sp. (CCUB 1739). M = molecular marker 100 bp DNA Ladder Cellco Biotec. NC = negative control. A full-length gel is presented in Supplementary Figure S1.

Primer sequences (Table 1) were compared against obtained sequences in GenBank, with BLAST (Basic Local Alignments Search Tool) analysis showing 100% homology of primers with sequences of isolates belonging to the species for which primers were designed. The primers targeting *EF1-α* gene were able to specifically amplify only isolates of *R. gossypii* and *R. pseudoglycines* (Fig. 5).

The primers sets RG-TEF-F/RG-TEF-R and RP-TEF-F/RP-TEF-R, specifically designed for the recognition of *R. gossypii* and *R. pseudoglycines*, respectively, successfully amplified a fragment of 900 bp from each *R. gossypii* isolate (Fig. 5) and an amplicon of 750 bp from each isolate of *R. pseudoglycines* (Fig. 5), respectively. The other fungal species (samples 10–20) did not have any fragment amplified.

Resultant amplicons were sequenced to confirm primer specificity. Comparison of their sequences with the target regions selected for primer design showed 100% homology, confirming the species-specificity of the primers. No cross-reactions were observed with the other species or genera tested.

**Table 1:** Primers selected for phylogenetic analysis and inter and intraspecific diversity analysis of *Ramulariopsis* isolates.

Locus ^1, 2, 3^	Primer Name	Sequence 5’ → 3’	Orientation	References	(Anealing/ Extension)
Actin (ACT) ^1^	ACT-512F	ATG TGC AAG GCC GGT TTC GC	Forward	Carbone & Kohn^26^	58°-25’’/72°-25’’
	ACT-783R	TAC GAG TCC TTC TGG CCC AT	Reverse	Carbone & Kohn^26^	58°-25’’/72°-25’’
BOX^2^	BOXA1R	CTA CGG CAA GGC GAC GCT GAC G	–	Martin et al.^27^	53°-60″/72°-8’
Elongation factor 1-α (EF1α)^1,3^	EF1F1^1^	TGC GGT GGT ATC GAC AAG CGT	Forward	Jacobs et al.^28^	54°-45’’/72°-45’’
	EF2R1^1^	AGC ATG TTG TCG CCG TTG AAG	Reverse	Jacobs et al.^28^	54°-45’’/72°-45’’
	RG-TEF-F^3^	GTCCAACCCACGACAGCGACATC	Forward	This study	59°-45’’/72°-30’’
	RG-TEF-R^3^	GTCTCCTTGATGATCTCGTTGTAGCGGT	Reverse	This study	59°-45’’/72°-30’’
	RP-TEF-F^3^	TCGCCTAATCCATCAAACCGACACC	Forward	This study	59°-45’’/72°-30’’
	RP-TEF-R^3^	GTCTCCTTGATGATCTCATTGTAGCGGT	Reverse	This study	59°-45’’/72°-30’’
	RP-TEF-F2^3^	CCGACACCGATACCAACAATAATACC	Forward	This study	59°-45’’/72°-30’’
	RG-TEF-R2^3^	GTCTCCTTGATGATCTCGTTGTAGCGGT	Forward	This study	59°-45’’/72°-30’’
	RG-TEF-R3^3^	GTATATTTTTCTCCCATTGTCACA	Reverse	This study	59°-45’’/72°-30’’
	RP-TEF-R2^3^	CCAACCCTCACGTGCAT	Reverse	This study	59°-45’’/72°-30’’
ERIC ^2^	ERIC1R	ATG TAA GCT CCT GGG GAT TCA C	Forward	Hulton et al.^29^	52°-60″/72°-8’
	ERIC2	AAG TAA GTG ACT GGG GTG AGC G	Reverse	Hulton et al.^29^	52°-60″/72°-8’
Glyceraldehyde-3-phosphate dehydrogenase (GAPDH) ^1^	GPD1^1^	CAA CGG CTT CGG TCG CAT TG	Forward	Berbee et al. ^30^	54°-45’’/72°-45’’
	GPD2_1_	GCC AAG CAG TTG GTT GTG C	Reverse	Berbee et al. ^30^	54°-45’’/72°-45’’
Histone H3 (HIS3) ^1^	CYLH3F	AGG TCC ACT GGT GGC AAG	Forward	Crous et al.^18^	60°-45’’/72°-30’’
	CYLH3R	AGC TGG ATG TCC TTG GAC TG	Reverse	Crous et al.^18^	60°-45’’/72°-30’’
IRAP ^2^	CIIRAP1	CGT ACG GAA CAC GCT ACA GA	–	Santos et al.^31^	57,5°-30″/72°-120”
	CIIRAP2	AAT AAC GTC TCG GCC TTC AG	–	Santana et al.^32^	55,4°-30″/ 72°-120”
	CIIRAP4	CTT TTG ACG AGG CCA TGC	–	Santos et al.^31^	54,9°-30″/ 72°-120”
ISSR ^2^	(CAG)^5^	CAG CAG CAG CAG CAG	–	Rodrigues et al.^33^	60°-45″/72°-90”
	(GA)^8^	GAG AGA GAG AGA GAG A	–	Andrea & Xitlali^34^	44°-45″/72°-90”
	(GACAC)^3^	GAC ACG ACA CGA CAC	–	Weising et al.^35^	48°-45″/72°-90”
	(TGTC)^4^	TGT CTG TCT GTC TGT C	–	Rodrigues et al.^33^	48°-45″/72°-90”
	(GTG)^5^	GTG GTG GTG GTG GTG	–	Gente et al*.*^36^	58°-45″/72°-90”
	(GACA)^4^	GAC AGA CAG ACA GAC A	–	Gente et al.^36^	50°-45″/72°-90”
	(GATA)^4^	GAT AGA TAG ATA GAT A	–	Gente et al.^36^	35°-45″/72°-90”
RNA polymerase II second largest subunit (RPB2) ^1^	RPB2-5f.	GAY GAY MGW GAT CAY TTY GG	Forward	Liu et al.^37^	54°-45’’/72°-45’’
	7cR	CCC ATR GCT TGY TTR CCC AT	Reverse	Liu et al.^37^	54°-45’’/72°-45’’
REP ^2^	REP1R-1	III ICG ICG ICA TCI GGC	Forward	Stern et al.^38^	44°-60″/72°-8’
	REP2-1	ICG ICT TAT CIG GCC TAC	Reverse	Stern et al.^38^	44°-60″/72°-8’
Universal primer N21 ^2^	N21	GGA TCC GAG GGT GGC GGT TCT	–	Bulat et al.^39^	55°-45″/72°-90”
Universal primer M13 ^2^	M13	GAG GGT GGC GGT TCT	–	Vassart et al.^40^	50°-45″/72°-90”
28S nrRNA (LSU) e Internal transcribed spacer (ITS) ^1^	V9G	TTA CGT CCC TGC CCT TTG TA	Forward	De Hoog & Ende^41^	53°-45’’/72°-45’’
	LR5	ATC CTG AGG GAA ACT TC	Reverse	Vilgalys & Hester^42^	53°-45’’/72°-45’’

^1^ Molecular marker employed for multigenic analysis; ^2^ Molecular marker employed to analyze genetic diversity. ^3^ Species-specific primers.

### Morphological characterization

**Figure 6:**
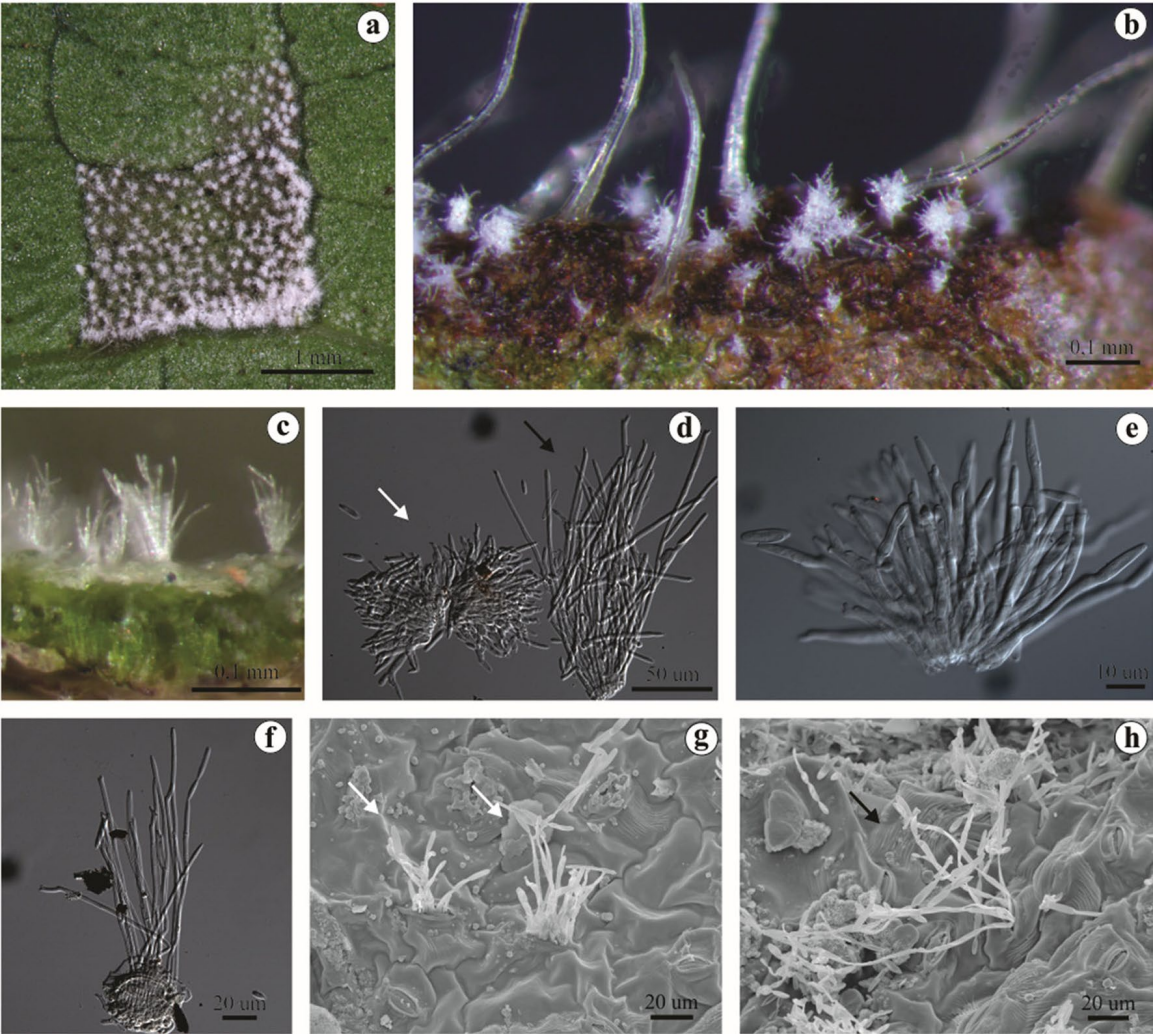
(**a–h**) *Ramulariopsis* spp. on leaves of *Gossypium hirsutum*. (**a**) Lesion with signs of the fungus on the leaf abaxial face. (**b**) Hyaline conidiophores of *R. gossypii* on the abaxial side of the leaves. (**c**) Hyaline conidiophores of *R. pseudoglycines* on the abaxial side of the leaves. (**d**) Hyaline conidiophores of *R. gossypii* (left) and *R. pseudoglycines* (right) viewed under light microscopy. (**e**) Fascicle of *R. gossypii* formed by conidiophores with presence of hyaline conidia. (**f**) Conidiophores of *R. pseudoglycines*. (**g**) Conidiophores of *R. gossypii* visualized in SEM. (**h**) Conidiophores of *R. pseudoglycines* visualized in SEM.

The morphological characteristics of the isolates belonging to *R. gossypii* and *R. pseudoglycines* in the concatenated tree matched well with the description of each species (Table 2). The long conidiophores of *R. pseudoglycines* are readily distinguished from the short conidiophores of *R. gossypii* by visualization under stereomicroscope or light microscopy. These morphological differences were recorded by SEM and are illustrated here for the first time (Fig. 6).

**Table 2:** Morphometric characteristics of *Ramulariopsis gossypii* and *R. pseudoglycines* on *Gossypium hirsutum* (Malvaceae).

SPECIES	Conidiophore µm	Conidia µm	Host
*R. gossypii*^1^	35‒40 × 3	18‒25 × 3‒4	*G. hirsutum*
*R. pseudoglycines*^2^	121–175 × 2	6.5–8 × 2.5–3	*G. hirsutum*
*R. gossypii* UB24343^3^	25‒44 × 2.5‒3.5	13‒19 × 3.5‒4.5	*G. hirsutum*
*R. pseudoglycines* UB24054^3^	74,5–138.5 × 2.5–3	11–21.5 × 3–3.5	*G. hirsutum*

^1^Spegazzini, 1886; ^2^Videira et al., 2016; ^3^Identified in this study by A.S.S. and D.B.P.

### Genetic characterization

**Figure 7:**
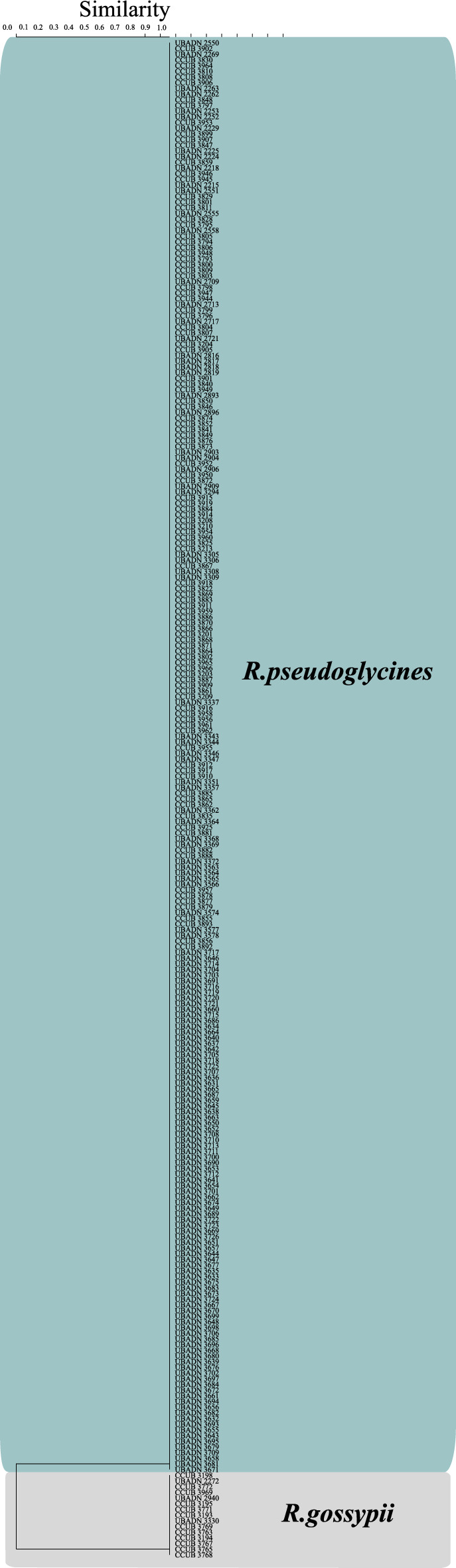
Dendrogram of 267 isolates of *Ramulariopsis*, generated by amplification with the ISSR (TGTC)^4^ primer, with the cutoff close to 70% of similarity. The genetic similarity pattern was generated by the UPGMA method, based on Jaccard’s coefficient.

A high interspecific polymorphism and a low intraspecific polymorphism were observed among *Ramulariopsis* isolates for all 14 markers. The dendrograms based on the binary matrix produced from the band patterns generated with all markers separately were used to analyze the interspecific diversity of *Ramulariopsis* (data not shown). The ISSR (TGTC)^4^ molecular marker was selected to estimate the interspecific diversity due to its´ simplicity for species discrimination. The resultant dendrogram of 267 isolates of *Ramulariopsis* revealed two distinct clades (Fig. 7) corresponding to the clades observed previously in the phylogenetic analysis.

### Genealogical network based on the *RPB2* gene

**Figure 8:**
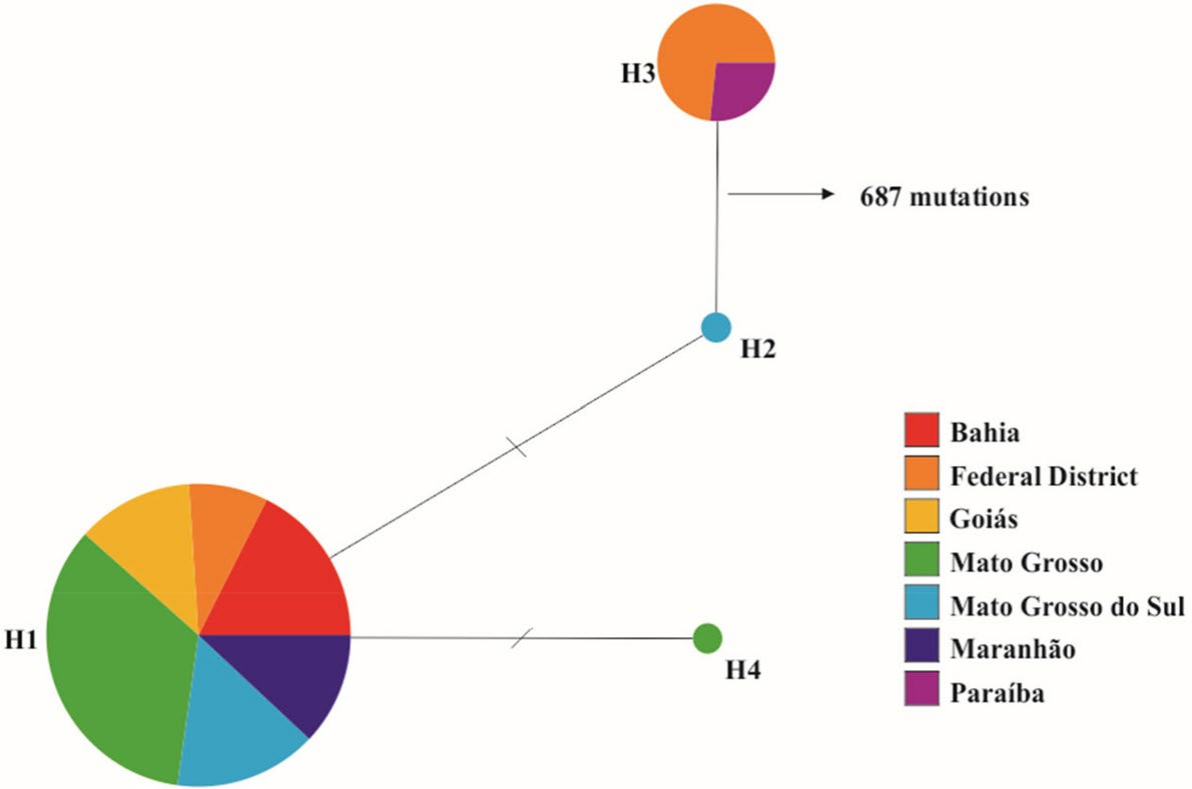
Haplotype network generated for *RPB2* sequences representing seven Brazilian states using Network. Each circle represents a distinct haplotype, proportional in size to its´ frequency in the sample. Hatch marks along the network branches indicate hypothetical mutational steps not detected in the dataset. Geographic origin of isolates from each haplotype by state is represented by colour.

Analysis of the genealogical network based on *RPB2* gene sequences revealed four distinct haplotypes among the 267 *Ramulariopsis* isolates (Supplementary table S1). The *RPB2* haplotype network (Fig. 8) distinguished two clear-cut clades corresponding to the clades observed in both the phylogenetic tree and the dendrogram. The first clade is restricted only to *R. gossypii* isolates from the Distrito Federal and Paraíba. The second clade is represented by isolates of *R. pseudoglycines* from multiple geographic regions, including all sampled locations. Two out of three *RPB2* haplotypes were represented by only one isolate of *R. pseudoglycines.* All isolates of *R. gossypii* (n = 15) grouped into a single haplotype (H3) while 250 out of the 252 isolates belong to the most frequent *R. pseudoglycines* haplotype (H1), indicating a strong prevalence of clonal populations.

## Discussion

Large-scale studies that investigate RLS etiology and the genetic variation of the causal agent are scarce in the literature. The centre of origin of cotton has not been determined, but the main centres of diversity are distributed among regions of Central America, Africa, Arabia and Australia^43^. In most of the countries within these regions, RLS is considered as a disease of secondary importance, in contrast to the present situation in Brazil.

Global cotton yield has been affected by *R. gossypii* since 1883^7^. Historically, this species is most widespread and economically important in Brazil, although it has been mostly identified based upon morphological data^4,15^. Although conidiophore length is useful for separating *Ramulariopsis* species, taxonomic expertise is required. Interestingly, several isolates previously putatively identified as *R. gossypii* were molecularly identified as either *R. gossypii* or *R. pseudoglycines*^15^. Comparison of *ITS* sequences of the *Ramulariopsis* isolates deposited in GenBank (nucleotide matrices and phylogenetic tree available in TreeBASE; study number S28159) revealed that *R. gossypii* and *R. pseudoglycines* were described in previous studies^17,44^. Isolates collected between 2017 and 2020 and molecularly characterized with sequences of the *ITS* region were identified as *R. pseudoglycines* in Brazil^25^.

Here, the molecular identification of the *Ramulariopsis* isolates causing RLS on cotton using a polyphasic approach confirmed the presence of *R. gossypii* and *R. pseudoglycines* in Brazil. In total, 252 out of 267 isolates were identified as *R. pseudoglycines* (94%), indicating that this species is the most widespread causal agent of cotton RLS in the Brazilian growing regions today. Additionally, isolates of *R. gossypii* were restricted to small and isolated farms located in the Distrito Federal and the state of Paraíba, while isolates of *R. pseudoglycines* were obtained from all sampled locations, and all extensive farms in the Brazilian Cerrado, which is the main cotton growing area in Brazil.

The clade assignments based on the concatenated-sequence tree (*RPB2*, *LSU*, *EF1-α*, *ITS*, *ACT*, and *HIS3*) were identical to those generated by *RPB2*-sequences trees, the *RPB2* haplotype network, the ISSR (TGTC)^4^ dendrogram, and the morphological comparisons. The most widely employed genomic regions for *Ramulariopsis* DNA-based identifications have, to date, been based upon *ITS* sequences, given both their high copy number and easy amplification, and the availability of universal primers. However, for various fungi, the *RPB2* molecular marker has been proposed in place of the *ITS* sequences, due to the lack of resolution in the latter and the potential presence of non-homologous *ITS* copies in individual fungal genomes^21^.

On a molecular level, *RPB2* sequences are recommended for accurate molecular-based identification of *Ramulariopsis*, given their universal application, speed, and the presumption that this molecular marker safely approximates taxonomic expertise. However, this technique is laborious, expensive, and requires time and knowledge of phylogenetic analysis for identifying species^45^. The desire for rapid, automated approaches, such as those obtained here using the ISSR (TGTC)^4^ primer and EF-α species-specific primers, indicates that the *RPB2* region can also potentially be applied for future simple and inexpensive diagnosis and detection assays.

This study showed that EF-α species-specific primers can be used for accurate molecular identification of *Ramulariopsis* isolates in Brazil, facilitating large-scale surveys of the distribution of species and monitoring of epidemics. Nevertheless, the primers developed here need to be validated for isolates collected in other countries. Prior to this study, the diversity among isolates of *Ramulariopsis* was verified through ERIC- and REP-PCR profiles for Brazilian isolates^23^ and RAPD profiles for Indian isolates^46^, although in both studies, only few isolates were examined, and accurate species identification was not achieved.

Considering the wide distribution of haplotype H1 of *R. pseudoglycines*, there is evidence for a predominant clonal lineage occurring in Brazil, indicating the existence of a highly efficient mechanism of dispersion over long distances. Although RLS caused by *R. gossypii* has been recognized for a long time, *R. pseudoglycines* seems to be firmly prevalent amongst the cotton-producing regions today. When comparing the morphology of *Ramulariopsis* specimens from earlier studies^4,16^, the morphological characteristics matched well with the description of *R. gossypii*.

Given the higher susceptibility of today’s main cotton genotypes and the frequency of fungicide applications, as well as the presence of the G143A substitution in the CYTB^25^ gene of *R. pseudoglycines*, which reduces sensitivity to strobilurins, we can hypothesize that extensive cultivation of a limited number of cotton genotypes over many successive growing seasons in the Cerrado has resulted in the increase in population size of *R. pseudoglycines*. An alternative hypothesis may be that *R. pseudoglycines* inhabited the native Cerrado vegetation and then spread to cotton plants. Clearly, this species has thus become the most important pathogen negatively impacting cotton production in Brazil today.

This is the first large-scale study that investigates the diversity of *Ramulariopsis* isolates associated with cotton. Validation of the EF-α species-specific primers as a tool to study the abundance and distribution of *Ramulariopsis* species will make it possible to carry out extensive RLS sampling studies worldwide. Finally, the correct identification of the RLS causal agent and its geographical distribution is essential for predicting resistance breakdown, guiding pesticide regimes and the development of disease-resistant genotypes.

## Methods

### Sampling and isolation

Cotton leaves showing typical symptoms of RLS were collected in the 2017–18 and 2018–19 growing seasons from 24 commercial fields in the Brazilian states of Bahia, Distrito Federal, Goiás, Maranhão, Mato Grosso, Mato Grosso do Sul and Paraíba (Fig. 1).

Fungal isolation into pure culture was carried out by the direct method^47^ in Petri dishes containing water-agar (WA) medium (20 g/L of agar). After 14 days of growth in WA, pure cultures were established by transferring a fragment of a hyphal tip to a new Petri dish containing malt extract (ME) medium (20 g/L malt extract and 20 g/L agar).

Isolates (Supplementary Table S1) were deposited in the Coleção de Culturas da Universidade de Brasília (CCUB; Brasília, Brazil) and stored at 18 ± 1 °C in sterile water^48^, 10% (v / v) sterile glycerol, and half potato-dextrose-agar (500 mL/L potato broth, 20 g/L agar and 20 g/L dextrose) slopes covered with sterile mineral oil.

### DNA extraction

Four mycelial discs (5 mm in diameter) were removed from the margin of 20-day-old pure cultures on ME and transferred to 250 mL conical flasks containing 50 mL of potato dextrose broth with the addition of streptomycin (500 mL/L potato broth, 20 g/L dextrose, 100 µg/mL streptomycin) and incubated at 25 °C, with a 12 h photoperiod.

After seven days growth, the developed mycelium was recovered on filter paper and transferred to 1.5 mL microtubes containing 30 µL of Tris–EDTA (TE) buffer, four metal beads (2.8 mm), and 600 mL of Nuclei Lysis Solution (Promega®). Total DNA extraction was performed using the Wizard Genomic DNA Purification Kit (Promega®) according to the manufacturer’s instructions. Total DNA preparations were analyzed via 1% agarose gel electrophoresis, stained with GelRed (Biotium R), and visualized under UV light. The DNA samples were stored at − 20 °C.

### Amplification and sequencing

Partial sequences of the gene encoding the second largest RNA polymerase II subunit (*RPB2*) were amplified using the specific PCR primers shown in Table 1. This genomic region was employed as the primary barcode for identification of *Ramulariopsis* species, given the high PCR success rate and easy alignment of the nucleotide sequences. To assign definite species demarcations for the *Ramulariopsis* isolates, partial nucleotide sequences of six nuclear genes, namely: 28S rRNA (*LSU*), the internal transcribed spacers of the ribosomal DNA (*ITS*), actin (*ACT*), elongation factor (*EF1-α*), glyceraldehyde-3-phosphate dehydrogenase (*GAPDH*), and histone H3 (*HIS3*) were obtained from representative isolates of different clades and locations preliminarily identified based on *RPB2* sequence data (Fig. 3). The amplification of the *GAPDH* gene of *R. pseudoglycines* isolates resulted in double bands from which the band with the correct estimated size was subsequently purified from agarose gels and sequenced by nanopore sequencing^49,50^. All primers employed are listed in Table 1, with respective annealing and extension parameters. The PCR mixtures consisted of 6.25 µL of MyTaq PCR Master Mix (2 ×), 0.3 µL of each primer (Table 1), 1 µL of genomic DNA (25 ng/µL) and 4.65 µL of ultrapure water. The cycling conditions were: Initial denaturation at 95 °C for 1.5 min, followed by 35 cycles at 95 °C for 20 s; annealing and extension according to Table 1 and a final extension at 72 °C for 5 min. PCR products were purified and bidirectionally Sanger-sequenced.

### Phylogenetic analyses

To determine to which *Ramulariopsis* species each isolate shared the highest nucleotide identity, the partial nucleotide sequences and the BLASTn algorithm were used to search the NCBI-GenBank nonredundant nucleotide database. A Bayesian phylogenetic tree was initially reconstructed using the *RPB2* sequences from the 267 isolates characterized here, and four representative isolates of *Ramulariopsis*. The ex-epitype CBS 141099 of *R. gossypii* was used as an outgroup. Also, phylogenetic trees were individually inferred from each genomic region analyzed here. Multiple sequence alignments were obtained with MAFFT v7^51^. Finally, Bayesian Inference (BI) and Maximum Likelihood (ML) phylogenetic trees were reconstructed using the concatenate data (RPB2, LSU, EF1-α, ITS, ACT, and HIS3). For BI, the best nucleotide substitution models were determined, for each partition, with MrModeltest. The CIPRES web portal^52^ was used to run MrBayes v3.2.1^53^. The Markov Chain Monte Carlo (MCMC) analysis was run with a total of 10 million generations, sampling every 1,000 generations. The convergence of the log likelihoods was confirmed using TRACER v1.7.1^54^. The first 25% of the sampled trees were discarded as burn-in, with the posterior probability (PP) values calculated with the remaining trees. The ML tree was reconstructed using RAxML v.8^55^, accessed through the CIPRES web portal^52^, assuming a general time reversible (GTR) nucleotide substitution model with a gamma (G) rate of heterogeneity, and 1,000 bootstrap replicates. Phylogenetic trees were visualized and edited in FigTree v1.4^56^ and Inkscape.

### Primer design and validation

The *EF1-α* sequences of *R. gossypii* and *R. pseudoglycines* were selected and aligned to enable searching for species-specific primers using Primer3 Plus and Primer-BLAST^57,58^. Additionally, divergent regions within the *EF1-α* sequences were selected for manual primers development. The specificities of the primer sequences were *in-silico*-tested prior to synthesis by searching similar DNA sequences on the NCBI database. Each specific primer was checked for the following parameters: primer length, primer melting temperature, GC content, GC clamp, primer secondary structures (hairpins, self-dimer, and cross dimer), repeats, runs and 3′ end stability^45^.

Seven species-specific primers were designed and screened against eight isolates from *R. gossypii* (n = 4) and *R. pseudoglycines* (n = 4). The screening also included ten fungal genera (*Aspergillus* sp., *Baudoinia* sp., *Cercospora* sp., *Colletotrichum* sp., *Fusarium* sp., *Lasiodiplodia* sp., *Phytophthora* sp., *Macrophomina* sp., *Talaromyces* sp., and *Trichoderma* sp.) that may occur on cotton plants or that can be found as contaminants. Each amplification was repeated at least twice in separate assays. The PCR parameters were the same as those mentioned above. Amplification products were visualized on 1.5% agarose gels stained with EtBr. After the initial screening, the validated primers were tested on all isolates.

### Light microscopy and SEM morphological characterization

For morphological characterization, specimens were initially observed with a Leica 205C stereomicroscope (Leica Biosystems, Nussloch GmbH, Nussloch, Germany). The microscopical characteristics were analyzed by mounting asexual structures in clear lactoglycerol, and 50 measurements for each morphological parameter were carried out at a magnification of × 1,000 using a Leica DM2500 light microscope equipped with a Leica DFC 490 digital camera, coupled to a computer containing the Leica Qwin-Plus software. The morphological characteristics of the isolates were compared with the description of *R. gossypii* and *R. pseudoglycines*^14,15^.

For examination on a scanning electron microscope (JOEL JSM-700 1F model), fragments of symptomatic dry leaves were fixed in 10 mm diameter copper stubs with double-sided carbon tape and coated with 25 mA gold, 1.10–2 mbar, for 2.5 min.

### Genetic characterization

Seventeen isolates of *R. gossypii* (n = 3) and *R. pseudoglycines* (n = 14) were subjected to characterization with different molecular markers (CIIRAP1-4, CIIRAP2-4, REP, ERIC, BOX, M13, N21, CAG5, GA8, GACAC3, TGTC4, GATA4, GTG5 and GACA4) which are typically highly polymorphic and useful in analysis of genetic variability of fungi^23,32,36^.

PCR amplifications were performed in a final volume of 12.5 µL: 6.25 µL of MyTaq PCR Master Mix (2 ×), 2.5 µL of primer, 1 µL of genomic DNA (25 ng/µL) and 2.75 µL of ultrapure water. Different volumes of primer were used for REP and ERIC (0.5 μl), and BOX (1 μl) molecular markers, with a final reaction volume again adjusted to 12.5 μL. The PCR conditions for each molecular marker are shown in the references listed in Table 1. Each amplification was repeated at least twice in separate assays.

The amplified products were evaluated as presence (1) or absence (0) of bands and recorded in a binary matrix. This matrix was added to the PAST3 software^59^, where the Jaccard similarity index was calculated for each combination of two samples. From the similarity index, dendrograms were constructed according to the unweighted pair group method with arithmetic mean (UPGMA).

### Genealogical network based on the *RPB2* gene

To characterize genetic diversity of *R. pseudoglycines* and *R. gossypii*, an analysis of haplotypes was performed using the RPB2 sequences of the 267 isolates. Haplotype identification was performed using the program DnaSP ver. 5.10.1^60^. A haplotype network to visualize the relationships among haplotypes representing seven Brazilian states was reconstructed using NETWORK 4.5.0.2 (Fluxus Technology Ltd.), with gaps and missing data excluded^61^.

### Ethics statement

The study complies with relevant institutional, national, and international guidelines and legislation. The activity of access to Genetic Heritage was registered with SisGen, compliance with law no. 13,123/2015 and its regulations, under permit number A724B5B dated 04/30/2018.

## Supplementary Information

Supplementary Information.

## Data Availability

The datasets generated in this study can be found in Genbank: MZ039858-MZ040124, MZ066658-MZ066720, and OM419332- OM419338; Treebase: S28159. The results obtained in this study are included in the contents of this report.
