# Supplementary material for: Ramularia leaf spot: PCR-based methods reveal widespread distribution of Ramulariopsis pseudoglycines and limited presence of R. gossypii in Brazil
Source: Sci Rep. 2023 Jun 17;13:9826. doi: 10.1038/s41598-023-33530-3 (PMC10276850; doi:10.1038/s41598-023-33530-3)
Supplement: Supplementary file 1 — Supplementary Information. [file 41598_2023_33530_MOESM1_ESM.docx]

**Supplementary Information**

**
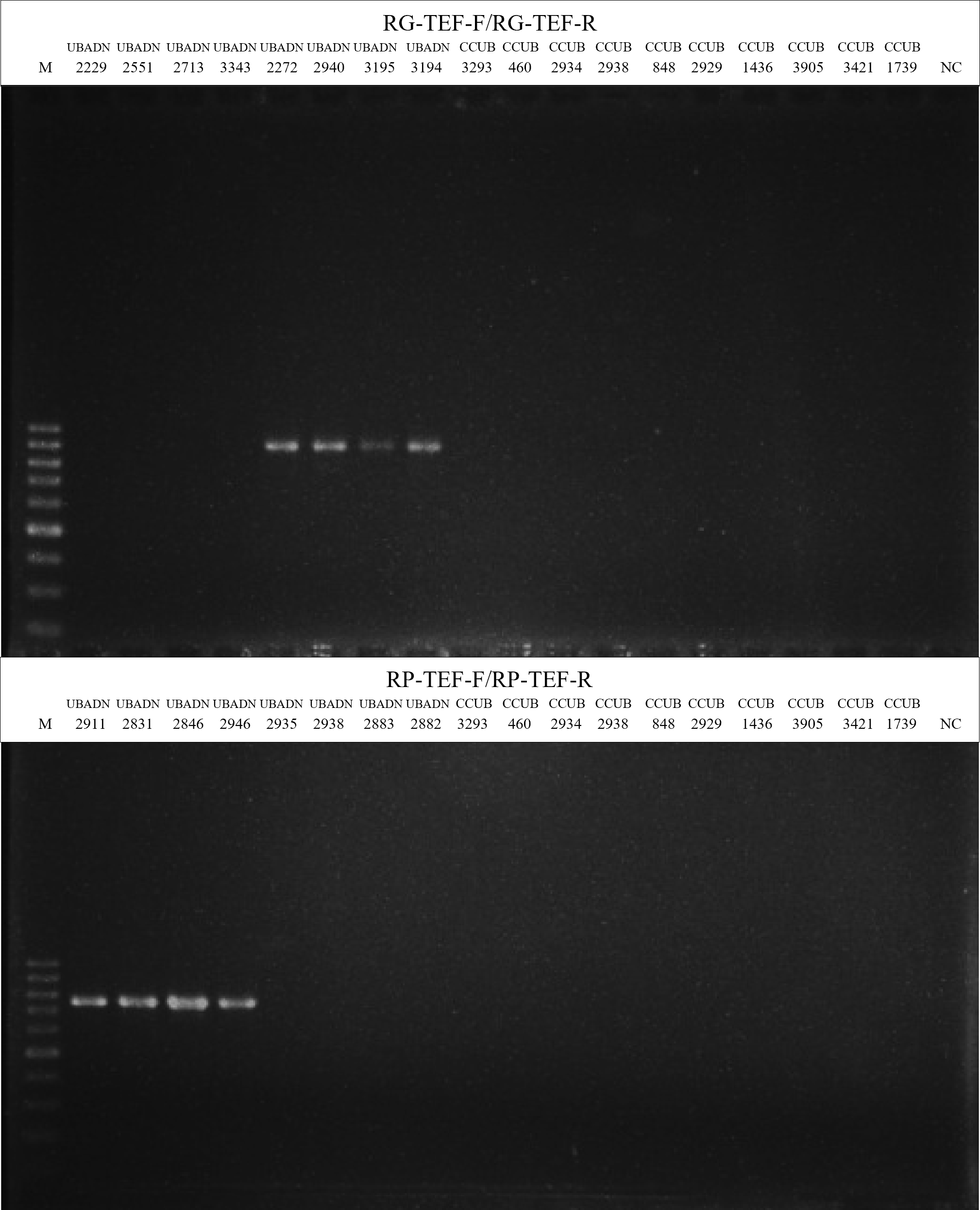
**

**Supplementary Figure S1.** Amplicons obtained using RG-TEF-F/RG-TEF-R and RP-TEF-F/RG-TEF-R primers and visualized on 1.5% agarose gels for isolates of *R. pseudoglycines* (UBADN 2229, UBADN 2551, UBADN 2713, UBADN 3343), *R. gossypii* (UBADN 2272, UBADN 2940, CCUB 3195, CCUB 3194), *Fusarium* sp. (CCUB 3293), *Colletotrichum* sp. (CCUB 460), *Talaromyces* sp. (CCUB 2934) and *Baudoinia* sp. (CCUB 2938), *Cercospora* sp. (CCUB 848), *Aspergillus* sp. (CCUB 2929), *Lasiodiplodia* sp. (CCUB 1436), *Macrophomina* sp. (CCUB 3905), *Trichoderma* sp. (CCUB 3421) and *Phytophthora* sp. (CCUB 1739). M = molecular marker 100 bp DNA Ladder Cellco Biotec. NC = negative control.

**Supplementary Table S1.** GenBank accession numbers of *RPB2* partial sequences of *Ramulariopsis* isolates included in this study.

| **Species** | **Isolate** | **Cotton genotype** | **City, State** | ***RPB2*** |
| --- | --- | --- | --- | --- |
| *R. gossypii* | CCUB 3767 | Not determined | Brasília, DF | MZ039867 |
| *R. gossypii* | CCUB 3768 | Not determined | Brasília, DF | MZ039868 |
| *R. gossypii* | CCUB 3769 | Not determined | Brasília, DF | MZ039871 |
| *R. gossypii* | CCUB 3193 | Not determined | Brasília, DF | MZ039858 |
| *R. gossypii* | CCUB 3771 | Not determined | Brasília, DF | MZ039869 |
| *R. gossypii* | CCUB 3194 | Not determined | Brasília, DF | MZ039859 |
| *R. gossypii* | CCUB 3195 | Not determined | Brasília, DF | MZ039860 |
| *R. gossypii* | CCUB 3763 | Not determined | Brasília, DF | MZ039866 |
| *R. gossypii* | CCUB 3765 | Not determined | Brasília, DF | MZ039872 |
| *R. gossypii* | UBADN 3330 | Not determined | Brasília, DF | MZ039870 |
| *R. gossypii* | CCUB 3772 | Not determined | Brasília, DF | MZ039864 |
| *R. gossypii* | UBADN 2272 | Not determined | Campina Grande, PB | MZ039861 |
| *R. gossypii* | CCUB 3969 | Not determined | Campina Grande, PB | MZ039865 |
| *R. gossypii* | UBADN 2940 | Not determined | Campina Grande, PB | MZ039862 |
| *R. gossypii* | CCUB 3198 | Not determined | Campina Grande, PB | MZ039863 |
| *R. pseudoglycines* | UBADN 3717 | Not determined | Balsas, MA | MZ040034 |
| *R. pseudoglycines* | UBADN 3703 | Not determined | Deciolândia, MT | MZ040035 |
| *R. pseudoglycines* | UBADN 3691 | Not determined | Deciolândia, MT | MZ040036 |
| *R. pseudoglycines* | UBADN 3716 | Not determined | Balsas, MA | MZ040037 |
| *R. pseudoglycines* | UBADN 3719 | Not determined | Campo Verde, MT | MZ040038 |
| *R. pseudoglycines* | UBADN 3720 | Not determined | Sapezal, MT | MZ040039 |
| *R. pseudoglycines* | UBADN 3721 | Not determined | Luis Eduardo Magalhães, BA | MZ040040 |
| *R. pseudoglycines* | UBADN 3660 | Not determined | Sapezal, MT | MZ040041 |
| *R. pseudoglycines* | UBADN 3715 | Not determined | Deciolândia, MT | MZ040042 |
| *R. pseudoglycines* | UBADN 3646 | Not determined | Costa Rica, MS | MZ040043 |
| *R. pseudoglycines* | UBADN 3714 | Not determined | Deciolândia, MT | MZ040044 |
| *R. pseudoglycines* | UBADN 3704 | Not determined | Costa Rica, MS | MZ040045 |
| *R. pseudoglycines* | UBADN 3686 | Not determined | Deciolândia, MT | MZ040046 |
| *R. pseudoglycines* | UBADN 3637 | Not determined | Cristalina, GO | MZ040047 |
| *R. pseudoglycines* | UBADN 3642 | Not determined | Costa Rica, MS | MZ040048 |
| *R. pseudoglycines* | UBADN 3705 | Not determined | Costa Rica, MS | MZ040049 |
| *R. pseudoglycines* | UBADN 3718 | Not determined | Deciolândia, MT | MZ040050 |
| *R. pseudoglycines* | UBADN 3725 | Not determined | Deciolândia, MT | MZ040051 |
| *R. pseudoglycines* | UBADN 3707 | Not determined | Deciolândia, MT | MZ040052 |
| *R. pseudoglycines* | UBADN 3636 | Not determined | Cristalina, GO | MZ040053 |
| *R. pseudoglycines* | UBADN 3631 | Not determined | Cristalina, GO | MZ040054 |
| *R. pseudoglycines* | UBADN 3634 | Not determined | Cristalina, GO | MZ040055 |
| *R. pseudoglycines* | UBADN 3664 | Not determined | Sapezal, MT | MZ040056 |
| *R. pseudoglycines* | UBADN 3640 | Not determined | Costa Rica, MS | MZ040057 |
| *R. pseudoglycines* | UBADN 3659 | Not determined | Sapezal, MT | MZ040058 |
| *R. pseudoglycines* | UBADN 3645 | Not determined | Costa Rica, MS | MZ040059 |
| *R. pseudoglycines* | UBADN 3638 | Not determined | Cristalina, GO | MZ040060 |
| *R. pseudoglycines* | UBADN 3663 | Not determined | Sapezal, MT | MZ040061 |
| *R. pseudoglycines* | UBADN 3650 | Not determined | Costa Rica, MS | MZ040062 |
| *R. pseudoglycines* | UBADN 3652 | Not determined | Sapezal, MT | MZ040063 |
| *R. pseudoglycines* | UBADN 3708 | Not determined | Cristalina, GO | MZ040064 |
| *R. pseudoglycines* | UBADN 3710 | Not determined | Cristalina, GO | MZ040065 |
| *R. pseudoglycines* | UBADN 3665 | Not determined | Sapezal, MT | MZ040066 |
| *R. pseudoglycines* | UBADN 3687 | Not determined | Deciolândia, MT | MZ040067 |
| *R. pseudoglycines* | UBADN 3713 | Not determined | Deciolândia, MT | MZ040068 |
| *R. pseudoglycines* | UBADN 3653 | Not determined | Sapezal, MT | MZ040069 |
| *R. pseudoglycines* | UBADN 3712 | Not determined | Deciolândia, MT | MZ040070 |
| *R. pseudoglycines* | UBADN 3641 | Not determined | Costa Rica, MS | MZ040071 |
| *R. pseudoglycines* | UBADN 3654 | Not determined | Sapezal, MT | MZ040072 |
| *R. pseudoglycines* | UBADN 3701 | Not determined | Cristalina, GO | MZ040073 |
| *R. pseudoglycines* | UBADN 3662 | Not determined | Sapezal, MT | MZ040074 |
| *R. pseudoglycines* | UBADN 3674 | Not determined | Balsas, MA | MZ040075 |
| *R. pseudoglycines* | UBADN 3649 | Not determined | Costa Rica, MS | MZ040076 |
| *R. pseudoglycines* | UBADN 3711 | Not determined | Cristalina, GO | MZ040077 |
| *R. pseudoglycines* | UBADN 3700 | Not determined | Costa Rica, MS | MZ040078 |
| *R. pseudoglycines* | UBADN 3690 | Not determined | Deciolândia, MT | MZ040079 |
| *R. pseudoglycines* | UBADN 3689 | Not determined | Deciolândia, MT | MZ040080 |
| *R. pseudoglycines* | UBADN 3726 | Not determined | Deciolândia, MT | MZ040081 |
| *R. pseudoglycines* | UBADN 3651 | Not determined | Costa Rica, MS | MZ040082 |
| *R. pseudoglycines* | UBADN 3657 | Not determined | Sapezal, MT | MZ040083 |
| *R. pseudoglycines* | UBADN 3644 | Not determined | Costa Rica, MS | MZ040084 |
| *R. pseudoglycines* | UBADN 3647 | Not determined | Costa Rica, MS | MZ039919 |
| *R. pseudoglycines* | UBADN 3677 | Not determined | Tasso Fragoso, MA | MZ040085 |
| *R. pseudoglycines* | UBADN 3635 | Not determined | Cristalina, GO | MZ040086 |
| *R. pseudoglycines* | UBADN 3633 | Not determined | Cristalina, GO | MZ040087 |
| *R. pseudoglycines* | UBADN 3722 | Not determined | Luis Eduardo Magalhães, BA | MZ040088 |
| *R. pseudoglycines* | UBADN 3723 | Not determined | Luis Eduardo Magalhães, BA | MZ040089 |
| *R. pseudoglycines* | UBADN 3669 | Not determined | São Desidério, BA | MZ040090 |
| *R. pseudoglycines* | UBADN 3724 | Not determined | Luis Eduardo Magalhães, BA | MZ040091 |
| *R. pseudoglycines* | UBADN 3667 | Not determined | Sapezal, MT | MZ040092 |
| *R. pseudoglycines* | UBADN 3670 | Not determined | São Desidério, BA | MZ040093 |
| *R. pseudoglycines* | UBADN 3699 | Not determined | Costa Rica, MS | MZ040094 |
| *R. pseudoglycines* | UBADN 3648 | Not determined | Costa Rica, MS | MZ040095 |
| *R. pseudoglycines* | UBADN 3698 | Not determined | Costa Rica, MS | MZ040096 |
| *R. pseudoglycines* | UBADN 3706 | Not determined | Balsas, MA | MZ040097 |
| *R. pseudoglycines* | UBADN 3675 | Not determined | Balsas, MA | MZ040098 |
| *R. pseudoglycines* | UBADN 3683 | Not determined | Deciolândia, MT | MZ040099 |
| *R. pseudoglycines* | UBADN 3673 | Not determined | Balsas, MA | MZ040100 |
| *R. pseudoglycines* | UBADN 3685 | Not determined | Deciolândia, MT | MZ040101 |
| *R. pseudoglycines* | UBADN 3639 | Not determined | Cristalina, GO | MZ040102 |
| *R. pseudoglycines* | UBADN 3676 | Not determined | Tasso Fragoso, MA | MZ040103 |
| *R. pseudoglycines* | UBADN 3702 | Not determined | Deciolândia, MT | MZ040104 |
| *R. pseudoglycines* | UBADN 3697 | Not determined | Costa Rica, MS | MZ040105 |
| *R. pseudoglycines* | UBADN 3684 | Not determined | Deciolândia, MT | MZ040106 |
| *R. pseudoglycines* | UBADN 3672 | Not determined | Balsas, MA | MZ040107 |
| *R. pseudoglycines* | UBADN 3661 | Not determined | Sapezal, MT | MZ040108 |
| *R. pseudoglycines* | UBADN 3694 | Not determined | São Desidério, BA | MZ040109 |
| *R. pseudoglycines* | UBADN 3696 | Not determined | Costa Rica, MS | MZ040110 |
| *R. pseudoglycines* | UBADN 3668 | Not determined | São Desidério, BA | MZ040111 |
| *R. pseudoglycines* | UBADN 3680 | Not determined | Balsas, MA | MZ040112 |
| *R. pseudoglycines* | UBADN 3656 | Not determined | Sapezal, MT | MZ040113 |
| *R. pseudoglycines* | UBADN 3655 | Not determined | Sapezal, MT | MZ040114 |
| *R. pseudoglycines* | UBADN 3643 | Not determined | Costa Rica, MS | MZ040115 |
| *R. pseudoglycines* | UBADN 3695 | Not determined | Costa Rica, MS | MZ040116 |
| *R. pseudoglycines* | UBADN 3679 | Not determined | Balsas, MA | MZ040117 |
| *R. pseudoglycines* | UBADN 3709 | Not determined | Cristalina, GO | MZ040118 |
| *R. pseudoglycines* | UBADN 3658 | Not determined | Sapezal, MT | MZ040119 |
| *R. pseudoglycines* | UBADN 3681 | Not determined | Balsas, MA | MZ040120 |
| *R. pseudoglycines* | UBADN 3671 | Not determined | Balsas, MA | MZ040121 |
| *R. pseudoglycines* | UBADN 3682 | Not determined | Balsas, MA | MZ040122 |
| *R. pseudoglycines* | UBADN 3632 | Not determined | Cristalina, GO | MZ040123 |
| *R. pseudoglycines* | UBADN 3693 | Not determined | Campo Verde, MT | MZ040124 |
| *R. pseudoglycines* | CCUB 3795 | Not determined | São Desidério, BA | MZ039920 |
| *R. pseudoglycines* | CCUB 3796 | Not determined | São Desidério, BA | MZ039937 |
| *R. pseudoglycines* | UBADN 2558 | Not determined | São Desidério, BA | MZ039877 |
| *R. pseudoglycines* | CCUB 3797 | Not determined | São Desidério, BA | MZ039908 |
| *R. pseudoglycines* | UBADN 2550 | Not determined | São Desidério, BA | MZ039917 |
| *R. pseudoglycines* | UBADN 2709 | Not determined | São Desidério, BA | MZ039932 |
| *R. pseudoglycines* | CCUB 3798 | Not determined | São Desidério, BA | MZ039933 |
| *R. pseudoglycines* | UBADN 2555 | Not determined | São Desidério, BA | MZ039923 |
| *R. pseudoglycines* | CCUB 3799 | Not determined | São Desidério, BA | MZ039936 |
| *R. pseudoglycines* | UBADN 2252 | Not determined | São Desidério, BA | MZ039903 |
| *R. pseudoglycines* | CCUB 3800 | Not determined | São Desidério, BA | MZ039930 |
| *R. pseudoglycines* | UBADN 2253 | Not determined | São Desidério, BA | MZ039905 |
| *R. pseudoglycines* | CCUB 3793 | Not determined | São Desidério, BA | MZ039929 |
| *R. pseudoglycines* | UBADN 2721 | Not determined | BA | MZ039940 |
| *R. pseudoglycines* | CCUB 3794 | Not determined | BA | MZ039926 |
| *R. pseudoglycines* | CCUB 3801 | Not determined | Barreiras, BA | MZ039921 |
| *R. pseudoglycines* | UBADN 2551 | Not determined | Barreiras, BA | MZ039918 |
| *R. pseudoglycines* | CCUB 3802 | Not determined | Luis Eduardo Magalhães, BA | MZ039989 |
| *R. pseudoglycines* | CCUB 3803 | DP1536B2RF | São Desidério, BA | MZ039931 |
| *R. pseudoglycines* | CCUB 3804 | DP1536B2RF | São Desidério, BA | MZ039938 |
| *R. pseudoglycines* | CCUB 3805 | DP1536B2RF | São Desidério, BA | MZ039924 |
| *R. pseudoglycines* | CCUB 3806 | DP1536B2RF | São Desidério, BA | MZ039927 |
| *R. pseudoglycines* | CCUB 3807 | DP1536B2RF | São Desidério, BA | MZ039939 |
| *R. pseudoglycines* | CCUB 3808 | FM975WS | São Desidério, BA | MZ039911 |
| *R. pseudoglycines* | CCUB 3809 | TMG81WS | São Desidério, BA | MZ039873 |
| *R. pseudoglycines* | CCUB 3810 | FM975WS | São Desidério, BA | MZ039913 |
| *R. pseudoglycines* | CCUB 3811 | FM975WS | São Desidério, BA | MZ039922 |
| *R. pseudoglycines* | CCUB 3828 | DP1536B2RF | Correntina, BA | MZ039925 |
| *R. pseudoglycines* | CCUB 3829 | DP1536B2RF | Correntina, BA | MZ039907 |
| *R. pseudoglycines* | UBADN 2263 | DP1536B2RF | Correntina, BA | MZ039910 |
| *R. pseudoglycines* | CCUB 3830 | FM975WS | Correntina, BA | MZ039914 |
| *R. pseudoglycines* | UBADN 2713 | Not determined | Alta Parnaíba, MA | MZ039935 |
| *R. pseudoglycines* | UBADN 2215 | Not determined | Alta Parnaíba, MA | MZ039900 |
| *R. pseudoglycines* | CCUB 3944 | TMG47B2RF | Alta Parnaíba, MA | MZ039874 |
| *R. pseudoglycines* | CCUB 3945 | TMG47B2RF | Alta Parnaíba, MA | MZ039901 |
| *R. pseudoglycines* | CCUB 3946 | DP1536B2RF | Alta Parnaíba, MA | MZ039894 |
| *R. pseudoglycines* | UBADN 2717 | DP1536B2RF | Alta Parnaíba, MA | MZ039878 |
| *R. pseudoglycines* | CCUB 3947 | TMG42WS | Alta Parnaíba, MA | MZ039934 |
| *R. pseudoglycines* | CCUB 3948 | TMG42WS | Alta Parnaíba, MA | MZ039928 |
| *R. pseudoglycines* | CCUB 3949 | TMG42WS | Alta Parnaíba, MA | MZ039947 |
| *R. pseudoglycines* | CCUB 3950 | TMG42WS | Alta Parnaíba, MA | MZ039962 |
| *R. pseudoglycines* | UBADN 2262 | FM975 | Balsas, MA | MZ039902 |
| *R. pseudoglycines* | UBADN 2906 | DP1536 | Balsas, MA | MZ039961 |
| *R. pseudoglycines* | CCUB 3201 | FM983 | Balsas, MA | MZ039879 |
| *R. pseudoglycines* | CCUB 3952 | DP1536B2RF | Balsas, MA | MZ039960 |
| *R. pseudoglycines* | CCUB 3953 | DP1536 | Balsas, MA | MZ039906 |
| *R. pseudoglycines* | UBADN 2893 | DP1536 | Balsas, MA | MZ039948 |
| *R. pseudoglycines* | UBADN 2904 | Not determined | Rio Verde, GO | MZ039959 |
| *R. pseudoglycines* | CCUB 3841 | Not determined | Rio Verde, GO | MZ039954 |
| *R. pseudoglycines* | CCUB 3846 | Not determined | Rio Verde, GO | MZ039950 |
| *R. pseudoglycines* | CCUB 3847 | Not determined | Rio Verde, GO | MZ039896 |
| *R. pseudoglycines* | CCUB 3850 | Not determined | Cristalina, GO | MZ039949 |
| *R. pseudoglycines* | UBADN 2269 | Not determined | Cristalina, GO | MZ039880 |
| *R. pseudoglycines* | CCUB 3848 | Not determined | Cristalina, GO | MZ039909 |
| *R. pseudoglycines* | CCUB 3849 | Not determined | Cristalina, GO | MZ039955 |
| *R. pseudoglycines* | CCUB 3852 | Not determined | Rio Verde, GO | MZ039953 |
| *R. pseudoglycines* | UBADN 2224 | Not determined | Formosa, GO | MZ039895 |
| *R. pseudoglycines* | CCUB 3859 | Not determined | Formosa, GO | MZ039893 |
| *R. pseudoglycines* | CCUB 3876 | Not determined | Rio Verde, GO | MZ039956 |
| *R. pseudoglycines* | CCUB 3203 | Not determined | Brasília, DF | MZ039881 |
| *R. pseudoglycines* | CCUB 3861 | Not determined | Planaltina, DF | MZ039994 |
| *R. pseudoglycines* | CCUB 3862 | Not determined | Planaltina, DF | MZ040010 |
| *R. pseudoglycines* | CCUB 3864 | Not determined | Planaltina, DF | MZ039988 |
| *R. pseudoglycines* | CCUB 3865 | Not determined | Planaltina, DF | MZ040009 |
| *R. pseudoglycines* | CCUB 3866 | Not determined | Planaltina, DF | MZ039985 |
| *R. pseudoglycines* | CCUB 3867 | Not determined | Planaltina, DF | MZ039975 |
| *R. pseudoglycines* | CCUB 3868 | Not determined | Planaltina, DF | MZ039986 |
| *R. pseudoglycines* | CCUB 3869 | Not determined | Planaltina, DF | MZ039979 |
| *R. pseudoglycines* | CCUB 3870 | Not determined | Planaltina, DF | MZ039984 |
| *R. pseudoglycines* | CCUB 3871 | Not determined | Planaltina, DF | MZ039987 |
| *R. pseudoglycines* | UBADN 2896 | 975WS | Planaltina, DF | MZ039951 |
| *R. pseudoglycines* | CCUB 3872 | 975WS | Planaltina, DF | MZ039963 |
| *R. pseudoglycines* | UBADN 2903 | 975WS | Planaltina, DF | MZ039958 |
| *R. pseudoglycines* | UBADN 2909 | 975WS | Planaltina, DF | MZ039964 |
| *R. pseudoglycines* | CCUB 3873 | 975WS | Planaltina, DF | MZ039957 |
| *R. pseudoglycines* | CCUB 3874 | 975WS | Planaltina, DF | MZ039952 |
| *R. pseudoglycines* | CCUB 3964 | FM975WS | Chapadão do Sul, MS | MZ039915 |
| *R. pseudoglycines* | UBADN 2229 | FM975WS | Chapadão do Sul, MS | MZ039904 |
| *R. pseudoglycines* | CCUB 3965 | FM975WS | Chapadão do Sul, MS | MZ039990 |
| *R. pseudoglycines* | CCUB 3966 | FM975WS | Chapadão do Sul, MS | MZ039991 |
| *R. pseudoglycines* | UBADN 3364 | Not determined | MT | MZ040013 |
| *R. pseudoglycines* | UBADN 2819 | Not determined | MT | MZ039945 |
| *R. pseudoglycines* | CCUB 3840 | Not determined | MT | MZ039875 |
| *R. pseudoglycines* | UBADN 2218 | IMA2106GL | Sapezal, MT | MZ039897 |
| *R. pseudoglycines* | CCUB 3899 | 44B2RF | Sapezal, MT | MZ039898 |
| *R. pseudoglycines* | CCUB 3901 | Not determined | Diamantino, MT | MZ039946 |
| *R. pseudoglycines* | CCUB 3902 | DIVERSAS | Diamantino, MT | MZ039916 |
| *R. pseudoglycines* | UBADN 2816 | FM975WS | Diamantino, MT | MZ039942 |
| *R. pseudoglycines* | CCUB 3204 | TMG44B2RF | Diamantino, MT | MZ039876 |
| *R. pseudoglycines* | CCUB 3905 | Not determined | Diamantino, MT | MZ039941 |
| *R. pseudoglycines* | CCUB 3906 | FM944GL | Diamantino, MT | MZ039912 |
| *R. pseudoglycines* | UBADN 2818 | FM944GL | Diamantino, MT | MZ039944 |
| *R. pseudoglycines* | UBADN 2225 | Not determined | Lucas do Rio Verde, MT | MZ039892 |
| *R. pseudoglycines* | CCUB 3907 | Not determined | Lucas do Rio Verde, MT | MZ039899 |
| *R. pseudoglycines* | CCUB 3960 | FM975WS | Chapadão do Sul, MS | MZ039971 |
| *R. pseudoglycines* | CCUB 3961 | FM975WS | Chapadão do Sul, MS | MZ039998 |
| *R. pseudoglycines* | CCUB 3962 | FM975WS | Chapadão do Sul, MS | MZ039999 |
| *R. pseudoglycines* | CCUB 3959 | FM975WS | Chapadão do Sul, MS | MZ039982 |
| *R. pseudoglycines* | CCUB 3958 | FM975WS | Chapadão do Sul, MS | MZ039997 |
| *R. pseudoglycines* | UBADN 3337 | FM975WS | Chapadão do Sul, MS | MZ039995 |
| *R. pseudoglycines* | UBADN 3343 | FM975WS | Chapadão do Sul, MS | MZ039882 |
| *R. pseudoglycines* | CCUB 3956 | FM975WS | Chapadão do Sul, MS | MZ039891 |
| *R. pseudoglycines* | UBADN 3308 | FM975WS | Chapadão do Sul, MS | MZ039976 |
| *R. pseudoglycines* | UBADN 3344 | FM975WS | Chapadão do Sul, MS | MZ040000 |
| *R. pseudoglycines* | CCUB 3954 | FM975WS | Chapadão do Sul, MS | MZ039970 |
| *R. pseudoglycines* | CCUB 3955 | FM975WS | Chapadão do Sul, MS | MZ040001 |
| *R. pseudoglycines* | UBADN 3346 | FM975WS | Chapadão do Sul, MS | MZ040002 |
| *R. pseudoglycines* | UBADN 3574 | FM975WS | Rio Verde, GO | MZ039883 |
| *R. pseudoglycines* | CCUB 3856 | FM975WS | Planaltina, DF | MZ040032 |
| *R. pseudoglycines* | CCUB 3855 | FM975WS | Planaltina, DF | MZ040028 |
| *R. pseudoglycines* | CCUB 3893 | FM906 | Campo Verde, MT | MZ040029 |
| *R. pseudoglycines* | UBADN 3577 | FM906 | Campo Verde, MT | MZ040030 |
| *R. pseudoglycines* | CCUB 3892 | FM906 | Campo Verde, MT | MZ040033 |
| *R. pseudoglycines* | UBADN 3578 | FM906 | Campo Verde, MT | MZ040031 |
| *R. pseudoglycines* | UBADN 3305 | FM975WS | Campo Verde, MT | MZ039973 |
| *R. pseudoglycines* | CCUB 3208 | FM975WS | Campo Verde, MT | MZ039885 |
| *R. pseudoglycines* | UBADN 3347 | FM975WS | Primavera do Leste, MT | MZ040003 |
| *R. pseudoglycines* | CCUB 3918 | FM975WS | Primavera do Leste, MT | MZ039977 |
| *R. pseudoglycines* | UBADN 3351 | FM975WS | Primavera do Leste, MT | MZ040007 |
| *R. pseudoglycines* | CCUB 3915 | FM975WS | Primavera do Leste, MT | MZ039966 |
| *R. pseudoglycines* | CCUB 3916 | FM975WS | Primavera do Leste, MT | MZ039996 |
| *R. pseudoglycines* | CCUB 3917 | FM975WS | Primavera do Leste, MT | MZ040005 |
| *R. pseudoglycines* | CCUB 3912 | FM975WS | Primavera do Leste, MT | MZ040004 |
| *R. pseudoglycines* | CCUB 3209 | FM975WS | Primavera do Leste, MT | MZ039886 |
| *R. pseudoglycines* | CCUB 3914 | FM975WS | Primavera do Leste, MT | MZ039969 |
| *R. pseudoglycines* | CCUB 3909 | FM975WS | Primavera do Leste, MT | MZ039993 |
| *R. pseudoglycines* | CCUB 3910 | FM975WS | Primavera do Leste, MT | MZ040006 |
| *R. pseudoglycines* | CCUB 3911 | FM975WS | Primavera do Leste, MT | MZ039981 |
| *R. pseudoglycines* | CCUB 3886 | FM954 | Campo Novo do Parecis, MT | MZ039983 |
| *R. pseudoglycines* | CCUB 3887 | FM954 | Campo Novo do Parecis, MT | MZ039992 |
| *R. pseudoglycines* | CCUB 3888 | FM954 | Campo Novo do Parecis, MT | MZ040019 |
| *R. pseudoglycines* | UBADN 3372 | FM954 | Campo Novo do Parecis, MT | MZ040020 |
| *R. pseudoglycines* | CCUB 3884 | FM954 | Campo Novo do Parecis, MT | MZ039968 |
| *R. pseudoglycines* | CCUB 3885 | FM954 | Campo Novo do Parecis, MT | MZ040008 |
| *R. pseudoglycines* | CCUB 3881 | FM954 | Campo Novo do Parecis, MT | MZ040015 |
| *R. pseudoglycines* | CCUB 3882 | FM954 | Campo Novo do Parecis, MT | MZ040018 |
| *R. pseudoglycines* | CCUB 3883 | FM954 | Campo Novo do Parecis, MT | MZ039980 |
| *R. pseudoglycines* | CCUB 3210 | FM954 | Campo Novo do Parecis, MT | MZ039887 |
| *R. pseudoglycines* | CCUB 3925 | FM975WS | Sapezal, MT | MZ040014 |
| *R. pseudoglycines* | UBADN 3362 | FM975WS | Sapezal, MT | MZ040011 |
| *R. pseudoglycines* | UBADN 3357 | FM975WS | Sapezal, MT | MZ039888 |
| *R. pseudoglycines* | UBADN 3369 | FM975WS | Sapezal, MT | MZ040017 |
| *R. pseudoglycines* | UBADN 3294 | FM975WS | Sorriso, MT | MZ039965 |
| *R. pseudoglycines* | UBADN 3306 | FM975WS | Sorriso, MT | MZ039974 |
| *R. pseudoglycines* | UBADN 3309 | FM975WS | Sorriso, MT | MZ039889 |
| *R. pseudoglycines* | CCUB 3919 | FM975WS | Sorriso, MT | MZ039967 |
| *R. pseudoglycines* | UBADN 3368 | FM975WS | São Desidério, BA | MZ040016 |
| *R. pseudoglycines* | CCUB 3835 | FM975WS | São Desidério, BA | MZ040012 |
| *R. pseudoglycines* | CCUB 3825 | FM975WS | Luis Eduardo Magalhães, BA | MZ039972 |
| *R. pseudoglycines* | CCUB 3822 | FM975WS | Luis Eduardo Magalhães, BA | MZ039978 |
| *R. pseudoglycines* | CCUB 3213 | FM975WS | Luis Eduardo Magalhães, BA | MZ039890 |
| *R. pseudoglycines* | UBADN 2817 | Not determined | Diamantino, MT | MZ039943 |
| *R. pseudoglycines* | UBADN 3564 | FM975WS | Chapadão do Sul, MS | MZ040021 |
| *R. pseudoglycines* | CCUB 3879 | FM975WS | Rio Verde, GO | MZ040027 |
| *R. pseudoglycines* | UBADN 3566 | FM975WS | Rio Verde, GO | MZ040023 |
| *R. pseudoglycines* | CCUB 3877 | FM975WS | Rio Verde, GO | MZ040026 |
| *R. pseudoglycines* | CCUB 3878 | FM975WS | Rio Verde, GO | MZ040025 |
| *R. pseudoglycines* | UBADN 3565 | FM975WS | Planaltina, DF | MZ040022 |
| *R. pseudoglycines* | UBADN 3563 | FM975WS | Planaltina, DF | MZ039884 |
| *R. pseudoglycines* | CCUB 3957 | FM975WS | Chapadão do Sul, MS | MZ040024 |

**Supplementary Table S2.** GenBank accession numbers of DNA sequences of *Ramulariopsis* isolates used in the multigenic analysis.

| **Species** | **Isolate** | **Cotton genotype** | **City, State** | ***LSU*** | ***ITS*** | ***ACT*** | ***EF1˗α*** | ***RPB2*** | ***HIS3*** | ***GAPDH*** |
| --- | --- | --- | --- | --- | --- | --- | --- | --- | --- | --- |
| *R. gossypii* | CBS 141099^ET^ | Not determined | Brazil | KX287243 | KX287540 | KX287823 | KX288102 | KX288702 | KX288991 |  |
|  | CPC 25909 |  |  |  |  |  |  |  |  |  |
| *R. gossypii* | CCUB 3193 | Not determined | Brasília, DF | MZ068040 | MZ068040 | MZ066662 | MZ066704 | MZ039858 | MZ066683 | OM419332 |
| *R. gossypii* | CCUB 3194 | Not determined | Brasília, DF | MZ068041 | MZ068041 | MZ066663 | MZ066705 | MZ039859 | MZ066684 | OM419333 |
| *R. gossypii* | CCUB 3195 | Not determined | Brasília, DF | MZ068039 | MZ068039 | MZ066661 | MZ066703 | MZ039860 | MZ066682 | OM419334 |
| *R. gossypii* | UBADN 2272 | Not determined | Campina Grande, PB | MZ068037 | MZ068037 | MZ066659 | MZ066701 | MZ039861 | MZ066680 | OM419335 |
| *R. gossypii* | UBADN 2940 | Not determined | Campina Grande, PB | MZ068038 | MZ068038 | MZ066660 | MZ066702 | MZ039862 | MZ066681 | OM419336 |
| *R. gossypii* | CCUB 3198 | Not determined | Campina Grande, PB | MZ068036 | MZ068036 | MZ066658 | MZ066700 | MZ039863 | MZ066679 | OM419337 |
| *R. pseudoglycines* | CPC 18241 | Not determined | Brazil | KX287245 | KX287542 | KX287825 | KX288104 | KX288704 | KX288993 |  |
| *R. pseudoglycines* | CBS 141100^T^ | Not determined | Brazil | KX287246 | KX287543 | KX287826 | KX288105 | KX288705 | KX288994 |  |
|  | CPC 18242 |  |  |  |  |  |  |  |  |  |
| *R. pseudoglycines* | CPC 20036 | Not determined | Togo | KX287244 | KX287541 | KX287824 | KX288103 | KX288703 | KX288992 |  |
| *R. pseudoglycines* | UBADN 2558 | Not determined | São Desidério, BA | MZ068043 | MZ068043 | MZ066665 | MZ066707 | MZ039877 | MZ066686 |  |
| *R. pseudoglycines* | UBADN 2717 | DP1536B2RF | Alta Parnaíba, MA | MZ068044 | MZ068044 | MZ066666 | MZ066708 | MZ039878 | MZ066687 |  |
| *R. pseudoglycines* | CCUB 3201 | FM983 | Balsas, MA | MZ068050 | MZ068050 | MZ066672 | MZ066714 | MZ039879 | MZ066693 |  |
| *R. pseudoglycines* | UBADN 2269 | Not determined | Cristalina, GO | MZ068042 | MZ068042 | MZ066664 | MZ066706 | MZ039880 | MZ066685 |  |
| *R. pseudoglycines* | CCUB 3203 | Not determined | Brasília, DF | MZ068051 | MZ068051 | MZ066673 | MZ066715 | MZ039881 | MZ066694 | OM419338 |
| *R. pseudoglycines* | CCUB 3204 | TMG44B2RF | Diamantino, MT | MZ068045 | MZ068045 | MZ066667 | MZ066709 | MZ039876 | MZ066688 |  |
| *R. pseudoglycines* | UBADN 3343 | FM975WS | Chapadão do Sul, MS | MZ068053 | MZ068053 | MZ066675 | MZ066717 | MZ039882 | MZ066696 |  |
| *R. pseudoglycines* | UBADN 3574 | FM975WS | Rio Verde, GO | MZ068056 | MZ068056 | MZ066678 | MZ066720 | MZ039883 | MZ066699 |  |
| *R. pseudoglycines* | UBADN 3563 | FM975WS | Planaltina, DF | MZ068055 | MZ068055 | MZ066677 | MZ066719 | MZ039884 | MZ066698 |  |
| *R. pseudoglycines* | CCUB 3208 | FM975WS | Campo Verde, MT | MZ068046 | MZ068046 | MZ066668 | MZ066710 | MZ039885 | MZ066689 |  |
| *R. pseudoglycines* | CCUB 3209 | FM975WS | Primavera do Leste, MT | MZ068052 | MZ068052 | MZ066674 | MZ066716 | MZ039886 | MZ066695 |  |
| *R. pseudoglycines* | CCUB 3210 | FM954 | Campo Novo do Parecis, MT | MZ068047 | MZ068047 | MZ066669 | MZ066711 | MZ039887 | MZ066690 |  |
| *R. pseudoglycines* | UBADN 3357 | FM975WS | Sapezal, MT | MZ068054 | MZ068054 | MZ066676 | MZ066718 | MZ039888 | MZ066697 |  |
| *R. pseudoglycines* | UBADN 3309 | FM975WS | Sorriso, MT | MZ068049 | MZ068049 | MZ066671 | MZ066713 | MZ039889 | MZ066692 |  |
| *R. pseudoglycines* | CCUB 3213 | FM975WS | Luis Eduardo Magalhães, BA | MZ068048 | MZ068048 | MZ066670 | MZ066712 | MZ039890 | MZ066691 |  |
